# Osteoarthritis Is a Low-Grade Inflammatory Disease: Obesity's Involvement and Herbal Treatment

**DOI:** 10.1155/2019/2037484

**Published:** 2019-11-04

**Authors:** Mustapha Zeddou

**Affiliations:** Team of Experimental Oncology and Natural Substances, Cellular & Molecular Immunopharmacology, Sultan Moulay Slimane University, Faculty of Sciences and Techniques, Beni Mellal, Morocco

## Abstract

Osteoarthritis (OA) is considered a major cause of disability around the globe. This handicapping disease causes important cartilage and bone alteration that is associated with serious pains and loss of joint function. Despite its frequent association with obesity, the aetiology of OA is not fully understood. In this review, the different aspects of OA and its correlation with obesity were analysed. Through examining different mechanisms by which obesity may trigger and/or exacerbate OA, we point out some relevant signalling pathways that may evolve as candidates for pharmacological drug development. As such, we also suggest a review of different herbal medicines (HMs) and their main compounds, which specifically interfere with the identified pathways. We have shown that obesity's involvement in OA is not only limited to the mechanical weight exerted on the joints (mechanical hypothesis), but also induces an inflammatory state by different mechanisms, including increased leptin expression, compromised gut mucosa, and/or gut microbiota disruption. The main signalling pathways involved in OA inflammation, which are associated with obesity, are protein tyrosine phosphatase 1B (PTP1B) and TLR4 or DAP12. Moreover, we also underline the contamination of plant extracts with LPS as an important factor to consider when studying HM's effects on articular cells. By summarizing recent publications, this review aims at highlighting newly established aspects of obesity involvement in OA other than the mechanical one.

## 1. Introduction

Osteoarthritis (OA) is a chronic joint syndrome that is characterised by a progressive breakdown of cartilage, osteophyte formation (abnormal bone growth), subchondral bone alteration (thickening), synovial inflammation and fibrosis, and articular cartilage degradation, which ultimately lead to loss of joint function. The main joints affected are the knees, hands, hip, and zygapophyseal joint. Data from the National Health Interview Survey indicate that an estimated 14 million people in the USA have symptomatic knee OA, including >3 million racial/ethnic minorities. Thus, OA is considered a major cause of disability and early retirement around the globe [1]. Indeed, the number of impairments in activities of daily living is 1.12–1.35 times greater among patients with OA than among healthy controls [2]. OA also has negative impacts on mental aspects of health, such as suicidal ideation [3] and perceived memory loss [4].

Although it is often associated with obesity, the aetiology of OA is not fully understood. In fact, few studies have deeply investigated the relationship between obesity and OA.

Furthermore, recent guidelines from the American Academy of Orthopaedic Surgeons from 2013 question the use of analgesics and intra-articular corticosteroids for OA treatment [5], whereas oral nonsteroidal anti-inflammatory drugs (NSAIDs) are conditionally recommended by the OA Research Society International and the American College of Rheumatology (ACR) [6, 7]. In addition, the long-term use of NSAIDs has been described to have many risks of side effects [8].

Thus, research in the field of herbal medicine has an important role in characterising new natural molecules that may target the signalling pathways involved in the onset of OA and its exacerbation. In this review, we deeply analyse the relationship between OA and obesity with a focus on the potential mechanisms and signalling pathways that could explain the correlation. Furthermore, we present a review of herbal medicines that precisely act on these identified signalling pathways as potential candidates for the development of natural anti-OA drugs.

## 2. Obesity Is a Determinant Factor of Low-Grade Inflammation Associated with Osteoarthritis

OA is often associated with obesity. A study on a cohort of 2597 participants in the United Kingdom showed a strong positive correlation between body mass index (BMI) and knee OA in men and women [9]. Similar results were found in American populations (African Americans and Caucasian Americans) [10]. Moreover, the ROAD study examined 1690 participants and showed that the prevalence and severity of knee OA were linked to metabolic syndrome and increased significantly with central obesity, high blood pressure, high triglycerides, low HDL cholesterol, and insulin resistance [11].

It seems almost intuitive that obesity affects the joints through mechanical loading, which increases proinflammatory factor expression, mainly by synovial fibroblasts (SVFs). Experimental evidence supports this “mechanical hypothesis.” Indeed, SVFs react to mechanical loading with enhanced expression and secretion of proinflammatory cytokines such as TNF-*α* and PG-E2 [12]. However, a review of the literature indicates that OA is more frequent in non-weight-bearing joints. In fact, OA of the hand is statistically associated with BMI and fat mass [13]. In addition, more OA of the grip was observed in obese patients [14]. Consequently, several hypotheses have been proposed to explain the degenerative state observed in non-weight-bearing joints.

### 2.1. Leptin Overexpression Is Detrimental in Osteoarthritis

Obesity has been declared by the American and Canadian Medical Associations as a chronic progressive disease and not just as risk factor for other diseases [15]. Furthermore, adipose tissue is no longer considered as a passive energy storage site, but a true endocrine “organ” with the ability to secrete adipokines such as leptin, resistin, and adiponectin. Adipocytes from obese patients express more proinflammatory cytokines (TNF-*α* and IL-6) than those from normal subjects [16]. The visceral fat of metabolically unhealthy obese patients contains more macrophages of the M2 phenotype (proinflammatory) than that of metabolically healthy obese subjects or lean subjects [17].

Moreover, the adipose tissue surrounding the articulation or even joint cells themselves could produce different adipokines such as leptin, which is believed to be detrimental in OA. Indeed, serum levels of leptin are independently and consistently associated with reduced cartilage thickness cross-sectionally and longitudinally [18]. Increased leptin levels have been shown in the synovial fluid in comparison with the serum in OA patients [19, 20]. In addition, patients with OA have significantly higher amounts of leptin in their synovial fluid than healthy controls [21].

Single-nucleotide polymorphism analysis has also demonstrated that the leptin gene and its receptor gene are associated with OA [22, 23]. By binding to the leptin receptor (Ob-Rb), increased leptin levels lead to elevated IL-6 expression through the JAK2/STAT3, p38 MAPK, or PI3K/Akt signalling pathways [24]. Leptin could also enhance the expression of other factors, such as IL-1, MMP9, and MMP13 [25, 26]. This suggests that leptin could induce the release of inflammatory cytokines and promote the progress of OA through cartilage damage [27].

The role of leptin in exacerbating the immune response in OA could be easily understood with regard to different roles of this adipokine as a potent regulator of the immune system. Indeed, leptin can increase NK cytotoxicity and the activation of granulocytes (neutrophils, basophils, and eosinophils) [28]. Additionally, it mediates the chemotaxis, infiltration, and survival of neutrophils [29]. This suggests that increased levels of leptin in obese patients may contribute to an overexpression of proinflammatory cytokines that participate in the detrimental effects observed in OA [27].

We previously demonstrated that synovial fibroblasts (SVFs) and chondrocytes drastically overexpress leptin when subjected to glucocorticoids (prednisolone) in vitro [30, 31]. Given the detrimental role of leptin, our observations raise more questions about the relevance of glucocorticoids in the treatment of OA, as well as the management of their side effects.

### 2.2. Osteoarthritis Is Associated with Obesity-Induced Disrupted Microbiota

For a long time, OA has been considered as a noninflammatory condition. However, nowadays, it is recognised that OA is associated with a low-grade inflammation [32] that is initiated by the innate immune system [33] and involves the macrophage-associated inflammatory response [34], activation of Toll-like receptor (TLR) pathways [33], and increased levels of soluble CD14, which is a TLR coreceptor protein that is released by activated proinflammatory macrophages [35]. TLRs are a group of membrane-associated pattern-recognition receptors that are overexpressed in OA patients. The abundance of activated macrophages in the knees of OA patients is correlated with the levels of sCD14 in their synovial liquid [35], which makes sCD14 a marker for innate immune system activation in OA.

One of the most plausible hypotheses to explain the obesity-associated inflammation observed in OA resides in the gut microbiota. The human intestine is populated by more than 1000 bacterial species, which mostly belong to Bacteroidetes, Firmicutes, and *Actinobacteria* which constitute the microbiota [36]. Accumulating evidence in humans and rodents suggests that the microbiota is a real “organ” that plays indispensable role in affecting host health metabolism and shaping systemic immunity.

The microbiota is also responsible for maintaining haemostasis of the gastrointestinal tract through regulating mucosal immunity, which keeps the intestinal ecosystem balanced. The microbiota achieves this equilibrium by preventing excessive immune response (to prevent its elimination) by making intestinal dendritic cells to be more tolerogenic. This in turn activates resident T-cell differentiation into the Th2 and Treg phenotype, which inhibit proinflammatory NF-*κ*B activation [37, 38].

Obesity has been shown to be associated with compromised gut mucosa, which is characterised by a disruption of the natural selectively permeable barrier between the circulation and intestinal lumen [39, 40]. Consequently, the whole bacteria and microbial products (endotoxins and lipopolysaccharides (LPS)) cross the systemic circulation in a process called microbial translocation. Microbial translocation is responsible for the innate immune system activation through LPS binding to TLRs. TLR activation results in NF-*κ*B translocation to the nucleus, followed by the transcription of inflammatory mediators associated with OA (IL-1, IL-6, and TNF-*α*) [41–43]. Furthermore, the normal gut microbial flora is also altered in parallel with the induction of chronic systemic inflammation in obese patients suffering from OA [44].

Different experiments that were mainly conducted on mice have indicated that different mechanisms may explain the raised serum level of LPS in obese patients. Findings have shown that a high-fat diet favours LPS-containing bacteria (Gram-negative) [39, 45]. Furthermore, insulin resistance, which is generally associated with obesity, leads to hyperglycaemia, resulting in increase of the gut mucosa [46, 47].

Finally, it is important to mention that circulating levels of leptin and its expression by adipose tissue are enhanced in response to LPS stimulation [48]. According to the mentioned roles of leptin in OA exacerbation, this result could be one more aspect of the dysbiosis of the gut microbiota with implications in OA.

Despite the clear association between obesity, microbiota alteration, and OA, the exact mechanism by which they are related is still unclear. However, several strategies have been proposed to overcome the complications through restoring bacterial balance. Therefore, the use of prebiotics, probiotics, or faecal transplantation has been proposed. Abundant literature has also described the use of herbal medicines in this issue.

### 2.3. Osteoarthritis and Insulin Resistance

In different studies, OA has shown a clear association with metabolic syndromes such as cardiovascular diseases (diastolic pressure), hyperglycaemia, and hypercholesterolemia [49, 50]. An association was also described between central fat mass, the risk of knee arthroplasty for osteoarthritis, and type II diabetes, independent of age and BMI [49]. In addition, a study on 1690 participants showed that the prevalence and severity of OA are associated with metabolic syndrome, which significantly increases with central obesity, diastolic pressure, high triglyceride levels, low HDL levels, and insulin resistance [11].

Moreover, OA and metabolic syndrome (especially insulin resistance) are often correlated. Indeed, nearly 47.3% of patients with type 2 diabetes are affected by OA [51]. Although a causal link has not yet been established, insulin resistance and OA coexistence may have different explanations, which includes the following: (1) OA and insulin resistance are both modulated by leptin levels [52]; (2) hyperglycaemia leads to increased gut mucosa permeability and absorption, which are implicated in OA [47]; and (3) insulin resistance is associated with elevated free fatty acids that may promote OA progression [53]. Thus, investigations of the molecular mechanism of the establishment or exacerbation of OA should focus on key signalling pathways that could be the link between OA and insulin resistance.

Toll-like receptor 4 (TLR4) is a modulator of innate immunity that contributes to both insulin resistance [54] and OA pathogenesis [55]. TLR activation results in the translocation of NF-*κ*B to the nucleus with the subsequent transcription of inflammatory mediators (IL-1, IL-6, and TNF-*α*), which are upregulated in OA joint tissues [41, 56]. Importantly, free fatty acids, which are abundant in obese patients, are ligands of the membrane-bound TLR4 and can promote inflammation [57, 58]. Furthermore, TLR4 knockout (KO) mice that were fed with high-fat diet were protected against articular cartilage damage despite becoming obese and glucose intolerant. In contrast, DAP12 KO mice, which are unable to inhibit TLR4 cytokine responses, developed accelerated cartilage catabolism [59–61].

DAP12 is a molecule with minimal extracellular and no signal-transducing elements other than a single ITAM, which recruits and activates Syk and ZAP70 in myeloid cells after tyrosine phosphorylation [62]. To our knowledge, the TLR4/DAP12 pathway has not yet been investigated in the context of medicinal plants. Many studies have published results about the effect of herbal medicines (HMs) on impaired glucose tolerance or insulin resistance [63]. It would be interesting to investigate the involvement of these HMs in the TLR4/DAP12 pathways. The use of animal models (TLR4 KO, DAP12 KO, or conditional KO mice) could be of great interest in this issue, in providing tools to finely dissect the mechanisms involved.

## 3. Herbal Remedies for OA Treatment

Given the growing problems related to OA around the world and the serious side effects of classical symptomatic treatment, there is a real need for new active molecules against OA. HM research has become an important focus in the scientific and medical communities as it allows for the identification of specific natural compounds that act on specific pathways. Further improvement of pharmacokinetic parameters of the identified compounds may result in effective drugs against OA. Numerous HMs have been studied to identify their mechanisms and biological effects on OA to replace corticotherapies and avoid its serious side effects [64].

### 3.1. Herbal Medicines Acting on Leptin Signalling Pathway

Natural molecules that act on obesity by inhibiting the leptin signalling pathway could be of great benefit for OA treatment at two levels. First, it could reduce BMI and thus lighten the burden on weight-bearing joints. Second, it could reduce proinflammatory cytokines that are promoted by leptin and are responsible for the detrimental effects observed in OA.

One of the well-studied leptin signalling pathways is the PTP1B pathway. PTP1B is an intracellular phosphatase that mediates the inactivation of both leptin and insulin pathways. This occurs through the dephosphorylation of specific phosphotyrosine residues (pTyr) of the leptin receptor (Ob-R) and insulin receptor (IR). The expression and activity of PTP1B are increased in patients who are surfing from obesity and insulin resistance [65–69]. PTP1B mutation is not lethal and does not induce any detectable side effects. Therefore, these observations may encourage pharmaceutical companies to work on identifying PTP1B inhibitors as potential drugs against obesity and diabetes [70].

Medicinal plants that have antidiabetic activity could also be tested for their effect on OA management since they also act as leptin pathway inhibitors. This plant-oriented drug strategy is safer, in regard to toxicity and side effects than available synthetic drugs. The main medicinal plants with effective PTP1B inhibitory effects are summarised in Table 1.

Given the wide and relatively unexploited source of bioactive compounds in marine environments, significant attention from the scientific community has been directed toward marine metabolites isolated from algae, sponges, invertebrates, sea urchins, seaweeds, soft corals, lichens, and sea grasses. More recently, compounds and extracts derived from marine organisms have been evaluated as PTP1B inhibitors. Some representative compounds belonging to the main classes with PTP1B inhibitory activity and isolated from marine organisms are summarised below.

Bromophenols have been isolated from different marine algae and have shown significant PTP1B inhibitory effects in vitro (red algae *Rhodomela confervoides* IC50 between 0.8 *μ*M and 4.5 *μ*M [96] and marine sponge *Lamellodysidea herbacea* IC50 from 0.9 *μ*M M to 1.7 *μ*M [97]).

Sesquiterpene quinones (dysidine and dysidavarone A) were isolated from the sea sponge *Dysidea* species. The PTP1B inhibitory activity exhibited by these two sesquiterpenes was significant (IC50 values of 6.7 *μ*M and 9.98 *μ*M, respectively) [98].

Stellettin G (isomalabaricane triterpene) was isolated from the Hainan sponge *Stelletta* species by Xue et al. This compound exhibits positive PTP1B inhibitory activity, with IC50 values of 4.1 *μ*M [99].

*Ecklonia stolonifera* and *Eisenia bicyclis* are brown algae, which contain eckol. These compounds exhibited variable PTP1B inhibitory activity in vitro. This activity was variable, with IC_50_ values ranging from 0.6 *μ*M to 55.5 *μ*M [100].

Aquastatin A is a fungal metabolite isolated from *Cosmospora species*. This compound received particular attention because of its low IC_50_ value against PTP1B enzyme (0.2 *μ*M) [101].

Diterpenes were isolated from *Sarcophyton trocheliophorum*. These compounds showed significant PTP1B inhibitory activity in vitro, with IC_50_ values ranging from 6.8 *μ*M to 27.1 *μ*M [102].

Fucoxanthin (carotenoid) isolated from *Phaeodactylum tricornutum*, *Eisenia bicyclis*, *Undaria pinnatifida*, and *Hijikia fusiformis*. A PTP1B inhibitory activity was observed with this compound with an IC_50_ value of 4.8 *μ*M [103].

*Hippospongia lachne*, a marine sponge found on Yongxing Island, exhibited a significant PTP1B inhibitory activity. This effect was mainly due to sesterterpenoids. The IC_50_ values were ranging from 5.2 *μ*M to 8.7 *μ*M [104].

Two compounds (brialmontin 1 and atraric acid) showing significant activity against PTP1B were isolated from *Lecidella carpathica*, an Antarctic lichen. IC_50_ values were 14 *μ*M and 51.5 *μ*M, respectively [105].

The PTP1B inhibitory activities exhibited by the ethanolic extract of the brown algae *S*. *serratifolium C*. Agardh was broad (IC_50_ 7.04 *μ*g/ml) [106].

Novel PTP1B inhibitors could be inspired by the key structures of the natural products. Despite being very potent in vitro, PTP1B inhibitors are handicapped by their low cell membrane permeability. The development process of PTP1B inhibitors should be focused together on binding affinity and inhibitory potency against intracellular PTP1B. This process is quit delicate. Indeed, most pTyr mimetics are highly polar, which makes them negatively charged at physiological pH, resulting in poor cell permeability and low oral bioavailability [107]. The challenge is to increase cell permeability through the design of small molecules with no charge or weaker charge. This issue seems quite difficult given that PTP active sites are highly conserved [108].

In this context, the search for natural compounds with PTP1B inhibitory activity may benefit from the *in silico* approach, which consists of high-throughput virtual screening (HTVS) followed by induced fit docking (IFD) against a target enzyme. Candidate compounds may be potent active PTP1B inhibitors if they satisfy all the *in silico* parameters (favourable docking score, glide energy, and hydrogen bond interactions with the active site residues) [109].

### 3.2. Herbal Medicines for Gut Microbiota Modulation

Disruption of the microbiota resulted in alteration of the population status of pathogenic microorganisms in the intestinal microbiota. Several therapeutic strategies have been proposed to manage this imbalance, which is also called dysbiosis. Probiotic therapy has been used for the treatment of inflammatory bowel diseases induced by disruption of the gut microbiota, and the results have been positive in terms of the protective and restorative effects on the bacterial gut population [110].

Various HM products are continuously being proposed for OA treatment, and some of them seem to have a direct effect on the gut microbiota. We present some of the HMs containing compounds belonging to the main classes, and that were recently described for their effects against microbiota dysbiosis.

*Ganoderma lucidum* is a medicinal Chinese plant shown to reduce Firmicutes-to-Bacteroidetes ratio, maintain intestinal barrier integrity, and reduce metabolic endotoxemia in mice. This effect is probably mediated by its high-molecular-weight polysaccharides [111].

Rat treatment with *Flos Lonicera*, a traditional eastern Asia herbal medicine, not only resulted in notable decrease in body and adipose tissue weights, but also regulated gut flora distribution and gut permeability, resulting in significant reduction of proinflammatory cytokines (TNF-*α*, COX-2, and IL-6) [112].

Berberine, an alkaloid from the benzylisoquinoline group generally extracted from different plants such as Berberis (*Berberis vulgaris*, *Berberis aristata*, *Mahonia aquifolium*, *Hydrastis canadensis*, *Xanthorhiza simplicissima*, *Coptis chinensis*, *Tinospora cordifolia*, *Argemone mexicana*, and *Eschscholzia californica*) could be considered as an effective agent for the treatment of obesity-associated OA as it contributes to gut microbiota restoration in mice with high-fat diets [113].

An ancient Chinese herbal formula was also used to restore bacterial profile disruption caused by a high-fat diet in mice. This formula is traditionally used in clinical practice and is prepared from five herbs, namely, *Artemisia capillaries* Thunb, *Polygonum cuspidatum Sieb*, *Curuma longa* L, *Hypericum japonicum*, and *Gardenia jasminoides Ellis.* The main changes induced in mice treated with this formula were the reduction of some opportunistic pathogens such as *Escherichia/Shigella* and the development of potentially beneficial bacteria like *Collinsella* [114].

Sangguayin is a Chinese traditional Herbal formula made by four dietary and medicinal plant components (leaf of *Morus alba* L., root of *Pueraria lobata*, root of *Dioscorea opposita* Thunb., and fruit of *Momordica charantia* L.). Sangguayin decoction was shown to reshape gut microbial structure and confer preventive effects against high-fat-diet-induced metabolic syndrome [115].

*Flos Abelmoschus manihot* is a traditional Chinese plant used as drug. It was shown to act on colitis by modifying gut microbiota composition. Indeed, *Flos Abelmoschus manihot* increased microbial diversity, and in particular, elevated the level of short-chain fatty acid (SCFA)-producing gut microbiota in colitic mice [116]. Similar effects were observed with asperlin, a marine-derived natural product that increased the Bacteroidetes-to-Firmicutes ratio [117].

*Luffa cylindrical* was shown, when supplemented in diet, to improve high-fat-diet (HFD)-induced gut microbiota dysbiosis. This effect was mediated by enhancing short-chain fatty acid (SCFA)-producing bacteria (e.g., *Blautia*). At long term, *Luffa cylindrical* was able to restore gut barrier damage and ovoid obesity development [118].

*Pandanus tectorius* fruit extracts rich in polyphenol were effective in preventing high-fat-diet (HFD)-induced gut microbiota dysbiosis. Indeed, *Pandanus tectorius* extracts enhanced the relative abundance of *Lactobacillus* and decreased the relative abundance of *Bacteroides* and *Alistipes* [119].

*Prunus domestica* Linn (Rosaceae), a functional food with multiple effects, has been investigated for its activity on gut microbiota of mildly hypercholesterolemic subjects. Prune essence concentrates intake during 4 weeks led to significant amelioration of the colony number of *Bifidobacterium* and *Lactobacillus*, but markedly lowered the colony number of *Clostridium perfringens* and *Escherichia coli* [120].

It has been shown that *Rhizoma Coptidis* alkaloids feeding positively modulated microbiota. Indeed, it significantly promoted the abundance of *Sporobacter termitidis*, *Alcaligenes faecalis*, and *Akkermansia muciniphila* in the gut of mice, whereas *Escherichia coli*, Desulfovibrio C21_c20, and *Parabacteroides distasonis* were suppressed [121].

A positive effect on gut microbiota was also observed using *Ligustrum robustum*. Indeed, increased faecal *Lactobacillus* and decreased Enterococci was observed in high-fat-diet-fed rats in vivo [122].

Saponins isolated from *Gynostemma pentaphyllum* have a beneficial effect on gut microbiota by reversing the host's inflammatory phenotype through increasing beneficial bacteria and decreasing sulfate-reducing bacteria [123].

*Gastrodia elata* is an old Chinese medicinal material. Experiments performed on mice demonstrated a positive regulation of this plant on gut microbiota. Indeed, fresh *Gastrodia elata* intake induced probiotics growth (Ruminiclostridium, Butyricicoccus, and Parvibacter) and pathogens decrease (*Escherichia*/*Shigella* and *Parasutterella*) [124].

Food supplementation with *Antrodia cinnamomea* has an anti-inflammatory effect on mice. This effect is mediated by the modulation of the composition of the gut microbiota, through reducing the Firmicutes*/*Bacteroidetes ratio and increasing the level of *Akkermansia muciniphila* and other bacterial species associated with anti-inflammatory properties [125].

It is very important to mention the crucial role of the gut microbiota in intestinal biotransformation of the different compounds found in HMs. Indeed, due to their poor solubility, many HMs are very little absorbed by the intestine. For instance, Feng et al. demonstrated that berberine, which exhibits a poor solubility, is converted by the microbiota into the absorbable but inactive dihydroberberine. However, once absorbed, dihydroberberine is oxidized back into berberine, which could exert its pharmacological effects [126]. Moreover, certain HMs are metabolized by the gut microbiota into metabolites that are more toxic than their precursors and could induce systemic acute toxicity after oral administration [127].

### 3.3. LPS Contamination of Plant Extracts Could Exacerbate Inflammation in OA

HMs are often described to have fewer side effects than conventional synthetic drugs. However, in previous personal observations, we noticed frequent LPS contamination of plant extracts. When plant extracts are used in vitro with SVFs, there was an overexpression of IL-6 and MMP-3. After careful investigation, we demonstrated that these increasing effects might be due to LPS contamination of the plant extracts, as blocking TLR4 with polymyxin B or TAK 242 was able to strongly reduce the induced IL-6 and MMP-3 expression (personal unpublished data). These findings raise an important issue to take into account when investigating the effects of a given plant. In fact, many studies could erroneously attribute increased expression of inflammatory mediators to a specific constituent of a plant if polymyxin B or TAK 242 controls are not included [79, 128, 129].

Blocking the effects of LPS in vitro could give an idea of the involvement of a given plant in an observed effect (or lack thereof). However, the situation is more complicated in vivo. Plant extracts are generally administrated to animals by oral gavage. Inhibiting the LPS effects by mixing plant extracts with a specific dose of polymyxin B before in vivo could raise the occurrence of side effects. For instance, dogs treated with polymyxin B developed hyperthermia, abdominal and facial flushing, and increased serum creatinine and urea nitrogen. Furthermore, rats that received doses of polymyxin B developed dyspnea, cyanosis, decreased physical activity, and ataxia. A solution to these in vivo side effects was found by using nonapeptide derived from polymyxin B, which controls anti-LPS activity but has less toxic side effects [130].

Moreover, the presence of LPS in plant extracts that are orally administrated to animals could be an exacerbating factor in cases of specific animal models, especially models of collagen-induced arthritis [131]. One could wonder if it would not be the case in humans suffering from OA and taking LPS-contaminated plant extracts as a remedy. In obese OA patients, a compromised gut mucosa may induce LPS translocation, which leads to a local and systemic increase of inflammatory mediators associated with OA.

Recently, the intra-articular injection of natural high-molecular-weight polysaccharide was proposed to supplement fluid in the knees of OA patients (Synvisc-One®, GO-ON®, and Hylan GF-20®). The injection mainly consists of sodium hyaluronate solution produced from chicken combs or obtained from bacterial fermentation processes and subsequent purification. Hyaluronate that has been produced in such a way should be absolutely of high grade and endotoxin free because of the high sensitivity of SVF to LPS. We observed that SVFs could respond to very small amounts of LPS (0.2 ng/ml) (personal unpublished data). Furthermore, many cases of granulomatous synovitis have been reported after intra-articular Hylan GF-20 injection [132, 133]. The contamination of plant extracts with LPS may be considered as one more aspect of intestinal bacteria and orally administrated HM interaction and should be taken into account.

## 4. Conclusion

OA is now considered as a low-grade inflammation disease, and its association with obesity is beyond the traumatism induced by the mechanical burden exerted on certain joints. Increased leptin levels in obese people have detrimental effects on the joints through the release of proinflammatory cytokines that promote the exacerbation of cartilage damage in OA. Obesity often goes together with insulin resistance or disruption of the gut microbiota.

Certain key signalling pathways identified in obesity or insulin resistance could be linked to OA. PTP1B, TLR4, and DAP12 seem to be excellent candidates for drug development. Studying the effects of HMs on these pathways may be very helpful to identify natural molecules that could be modified for better pharmacokinetic parameters. HMs with significant effects on restoring microbiota equilibrium may also help to develop antiosteoarthritis drugs.

## Figures and Tables

**Table 1:** Medicinal plant-isolated compounds with PTP1B inhibitory effect.

Plant name	Medicinal purpose	Compounds with PTP1B inhibitory effect	IC_50_
*Artemisia dracunculus L.* (*Tarragon*)	Insomnia, skin wounds, irritation, allergic rashes and dermatitis, and antibacterial and antifungal activity	20,40-Dihydroxy-4-methoxydihydrochalcone and davidigenin [71]	—
*Panax notoginseng* (Chinese ginseng)	Antiageing, antitumor, immunostimulating, blood circulation	Dammarane-type triterpenoids [72]	26,265 *μ*M
*Erythrina addisoniae* (Leguminosae)	Pain, skin tumors apoptosis, hepatitis, and rheumatic disorders	Prenylated isoflavonoid 2-arylbenzofurans [73]	4.6 to 24.2 *μ*M
*Erythrina mildbraedii*	Anti-inflammatory activity, antiendocrine cancer cells	Prenylated flavonoids [74]	5.3 to 42.6 *μ*M
*Cladophora socialis*	Antimicrobial, antioxidant	Vanillic acid derivative [75]	3.7 *μ*M
*Paeonia lactiflora*	Pain, rheumatoid arthritis, lupus erythematosus hepatitis, dysmenorrhea, muscle cramping, spasms	1,2,3,4,6-Penta-O-galloyl-d-glucopyranose [76]	4.8 *μ*M
*Morinda citrifolia*	Antibacterial, antiviral, antifungal, diabetes, high blood pressure, cancer	Episesamin 2,6-dicatechol, pinoresinol, lirioresinol, americanin A, ursolic acid, and arteminorin D [77]	4.12 to 21.86 *μ*M
*Nigella glandulifera*	Inflammation, headache, fever, asthma, cough, bronchitis, rheumatism, diabetes, eczema, influenza	Glycybenzofuran and licocoumarone [78]	6.44 to 16.85 *μ*M
*Glycyrrhiza uralensis*	Peptic ulcers, hepatitis, liver and respiratory disease, Alzheimer, chronic fatigue, cancers	Flavonoid dimers licoagrone and licoagrodin [79]	6.0 to 11.5 *μ*M
*Rhododendron brachycarpum G. Don*	Hypertension, headache, and rheumatoid arthritis	Ursolic acid, rhododendric acid, corosolic acid, and 23-hydroxyursolic acid [80]	3.1 to 7.4 *μ*M
*Weigela subsessilis*	Pain and allergic syndromes	24-norursane triterpenes, ilekudinol A and B [81]	5.3 and 29.1 *μ*M
*Nitraria sibirica*	Hypotensive, anti-inflammatory renal injury	Benzyl-O-*β*-d-glucopyranoside and (3S,5R,6R,7E,9S)-megastigmane-7-ene-3-hydroxy-5,6-epoxy-9-O-*β*-d-glucopyranoside [82]	6.97 and 11.76 *μ*M
*Sophora flavescens*	Liver fibrosis treatment, anti-inflammatory, antiviral	Flavanone fused with a dihydrochalcone skeleton [83]	0.33 to 0.35 *μ*M
*Ficus racemosa*	Anti-inflammatory, wound healing	Soderrone, derrone, alpinumisoflavone, mucusisoflavone [84]	22.7, 12.6, 21.2, and 2.5 *μ*M
*Melaleuca leucadendron* (Myrtaceae)	Tranquilizing, sedating, and pain-relieving	Betulinic acid and ursolic acid [85]	1.5 and 2.3 *μ*g/mL
*Veratrum nigrum*	Hypertension, stroke, and excessive phlegm	Jervine-3-yl formate and veratramine-3-yl acetate [86]	11.3 and 4.7 *μ*M
*Camellia japonica L.* (Theaceae)	Anti-inflammatory and antiviral activity, platelet aggregating, gastroprotective	3b,16a,17b-trihydroxy-olean-12-ene, 3b-hydroxy-olean-11,13(18)-diene-28-oic acid [87]	3.77 to 6.40 *μ*M
*Psoralea corylifolia*	Gynecological bleeding, vitiligo, and psoriasis	Psoralidin [88]	9.4 *μ*M
*Myristica fragrans*	Antimicrobial, anti-inflammatory	Meso-dihydroguaiaretic acid and otobaphenol [89]	19.6 and 48.9 *μ*M
*Cyclocarya paliurus*	Hypertension, antimicrobial, and colon health-promoting	4-Di-O-*β*-D-glucopyranoside [90]	10.50 *μ*M
*Ligularia fischeri*	Bacterial infections, rheumatism, bronchitis, asthma	Sesquiterpene [91]	1.34 *μ*M
*Cichorium glandulosum*	Liver and gallbladder disease	Lactucin [92]	≈1 *μ*M
*Saussurea lappa*	Rheumatism, headache, stomach ache, and throat infection	Mokko lactone and dehydrocostuslactone [93]	1.41 and 6.51 *μ*g/mL
*Salvia miltiorrhiza*	Liver disorders, vascular, menstrual, and blood circulation systems	Isotanshinone, dihydroisotanshinone, and isocryptotanshinone [94]	11.4, 22.4, and 56.1 *μ*M
*Aegiceras corniculatum*	Painful arthritis, inflammation, asthma	Falcarindiol [95]	9.15 *μ*M
